# Treatment without consent in adult psychiatry inpatient units: a retrospective study on predictive factors

**DOI:** 10.3389/fpsyt.2023.1224328

**Published:** 2023-08-10

**Authors:** Giulia Meroni, Othman Sentissi, Stefan Kaiser, Alexandre Wullschleger

**Affiliations:** Division of Adult Psychiatry, Department of Psychiatry, Geneva University Hospitals, Geneva, Switzerland

**Keywords:** coercion, forced medication, law and psychiatry, inpatient psychiatric care, psychosis

## Abstract

**Background:**

Coercion is one of the most important challenges in mental health. In Switzerland, forced medication can be applied during an emergency (Art. 435 of the Civil Code) or over a longer period in case of endangerment of others or oneself (Art. 434). We aimed to analyze the predictors of this specific treatment without consent.

**Methods:**

Forced medication prescriptions in the Division of Adult Psychiatry of the Geneva University Hospitals between 2018 and 2021 were retrospectively analyzed. Medication under Article 434 was the main outcome variable. Age, gender, admission mode, main diagnosis, and the Health of the Nation Outcome Scales (HoNOS) score at admission were considered as potential predictors. *T*-test and Pearson’s chi-square test were used to compare continuous and categorical variables. A logistic regression was performed to find significant predictors of forced medication.

**Results:**

Seventy-one out of 4,326 inpatients were subjected to forced medication under Art. 434. HoNOS global scores at admission were not significantly different in the forced medication group compared to the control group. Aggressive behavior was lower in the former at the univariate level. Forced medication was associated at the multivariate level with female gender, involuntary admission, and psychosis.

**Conclusion:**

Women suffering from psychosis are more at risk of receiving involuntary and repeated medication. The risk of deterioration in psychosocial functioning or behavioral disorganization seems to be the main argument for this coercive measure. Future studies should focus on the patient’s perception of this coercion to prevent it and improve adherence to care. Follow-up after discharge might be useful to evaluate a long-term benefit.

## 1. Introduction

The use of coercive measures remains an ethical and clinical challenge in psychiatry (1). Coercion involves legal, human rights-related, and ethical issues and should thus be a central concern for mental health services (2). Coercive measures such as forced medication, seclusion or mechanical restraint can have serious physical and psychological consequences on patients, and often go with feelings of abuse, punishment, or humiliation and anxiety (3–6). Coercive measures and coercive practices in general negatively affect the therapeutic relationship is therefore impacted by coercive measures. Patients describe feelings of anger against caregivers, especially if the medical decision is not explained to them (7). Caregivers consider coercion partly as therapeutic and, at the same time, as causing negative emotions (8). According to a review by Chieze et al. (3), the rate of patients facing coercive measures in psychiatry varies between 0.4 and 66%, with remarkable differences depending on the country’s care habits.

The use of coercion considerably differs between countries and institutions, as highlighted among other works by the EUNOMIA project (9). More specifically, procedures involving forced medication are not homogeneous between these countries. In general, relatively few studies are focused on the use of forced medication (10). A comparison of legislations regarding forced medication among four representative countries (England, Germany, France, and Italy) near Switzerland geographically and culturally shows how heterogenous this coercive measure is implemented in practice.

In Switzerland, two different legislative articles regulate patients’ treatment at the hospital, depending on whether there is a state of acute emergency or not. The administration of treatment without consent - not necessarily in an emergency - is regulated by the Article 434 of the Civil Code. It concerns the repeated administration of medication to treat a pathology and not only to manage acute agitation. Rather, Article 435 of the Civil Code regulates the treatment in emergencies. This means that a unique dose of medication can be administrated immediately, whether the protection of the person or others demands it. In general, according to the law, during involuntary hospitalizations, a patient may receive forced medication if their consent is lacking in the three following circumstances. First, the lack of treatment could seriously endanger the health of the person concerned, as well as the life and integrity of others. Second, the patient does not have the ability to discern the need for treatment. Third, there are no less invasive measures available to limit the danger. However, as explained above, there is a real difference between an isolated emergency episode (single emergency treatment regulated by Art. 435) and a recurrent medication without consent (regulated by Art. 434). In the second situation, the patient has the legal right to appeal to the Civil Court (French: *TPAE, Tribunal de Protection de l’Adulte et de l’Enfant*). The treatment can be started immediately, independently of the patient’s appeal. This treatment is limited to the inpatient setting.

Legislations and practices regarding forced medication vary between European countries. In England, patients hospitalized against their will are forced to take treatment for 3 months if there is a risk of deterioration of their health or if they represent a danger to themselves or others. If the patient is still opposed to treatment after ending the medication period, the physician must refer to the Care Quality Commission. Another doctor (Second Opinion Appointed Doctors) has to evaluate the patient following the patient’s request (11).

In Germany, even if a patient is hospitalized against his will, a civil court must rule over the therapeutic project, which must be presented in detail by the doctor in charge of the patient. An expert psychiatrist’s opinion is also needed. Usually, forced medication is only allowed for a maximum of 2 weeks. If the period is to be prolonged, the court must be consulted again (12, 13).

In France, a patient who is hospitalized against his will is obliged to receive medications for a renewable period of 1 month. If the duration of care exceeds a continuous period of 1 year from the time of admission, the maintenance of the medication depends on a medical evaluation conducted by a panel of experts. A patient who is « fully hospitalized » (French: *en hospitalisation complète*) cannot refuse the treatment. This means that the patient needs to stay at the hospital day and night. It differs from part-time hospitalization (French: *hospitalisation à temps partiel*), which means that a specific program is established with the patient, who can stay alternately at home or at the hospital (14).

In Italy, during forced hospitalizations, the medication is decided by the psychiatrist and is compulsory for the patient during the entire time of hospitalization (15).

To sum up, among these five countries, only Switzerland and Germany make a real distinction between forced hospitalization and medication without consent, which are practices regulated by two different laws. In England, France, and Italy, patients forced into hospital care are obliged to receive medical treatment. In Switzerland, the Civil Court intervenes if the patient appeals against the forced medication; however, in Germany, the Civil Court is always involved in the decision.

Numerous publications focus on the issue of coercion, but only a few of these target the use of forced medication alone, and even less focus on forced non-emergency medication (16). A literature review was conducted by Jarrett and al. to identify a demographic and clinical profile of patients treated against their will (17). The authors also aimed to investigate the experiences of patients and caregivers in this context. According to this review, people who tend to receive forced treatment are in their 30 and suffer from bipolar disorder, schizophrenia, or other psychotic disorders. The main cause of treatment without consent seems to be hetero-aggressive behavior. However, this review does not make a distinction between emergency and non-emergency medication. Probably because of quite different international laws, there are few studies available on the subject. It seems clinically interesting to distinguish this treatment from forced emergency treatment, especially in terms of consequences for the patient. It would also be useful to investigate the differences from other kinds of coercive measures. There is currently no information in Switzerland about the further course of these patients treated against their will under Article 434.

The present study addresses the use of treatment against the will of the patient in non-urgent situations. The goal of our research is to investigate whether certain socio-demographic and clinical factors could predispose to treatment without consent. Furthermore, we aim to describe the way this coercive measure is applied, as well as its outcome. This study should help initiate a clinical reflection on this understudied form of coercion.

## 2. Materials and methods

### 2.1. Participant selection

This study analyses retrospective data. We collected patient sociodemographic and clinical characteristics, duration of hospital stays, and forced medication prescriptions from patients’ electronic records. The data were anonymized. The present study is a secondary analysis of data gathered in a retrospective analysis of coercive measures in our division. The project was approved by the local ethics committee (*Commission Cantonale d’Ethique de la Recherche du Canton de Genève*; No. 2018–00988).

Patients hospitalized in the Division of Adult Psychiatry at the University Hospital of Geneva between January 1, 2018, and December 31, 2021, were included. The Division includes six inpatient wards providing acute psychiatric care to patients aged between 18 and 65 years without any diagnostic distinction. Each ward has between 14 and 18 beds and one seclusion room. Concerning the staff, 3–5 nurses work in each unit at the same time, and there are 3–4 physicians during the working day. Starting from 7 p.m. till 8 a.m., there are only two nurses in each unit and one doctor for the whole hospital.

### 2.2. Data collection

The prescription of treatment without consent according to the Article 434 of the Civil Code, i.e., forced treatment over a longer period in inpatient settings, outside of emergencies, was defined as the main (dependent) outcome variable. The number of prescriptions was, therefore, automatically extracted from patients’ general medical records. Patients who received emergency forced medication or were not subject to forced medication were included in the comparison group.

#### 2.2.1. Clinical and socio-demographic data

Following patient-related variables (demographic items) and clinical factors were studied: age, gender, the issuer of the hospitalization decision (emergency department, private doctor etc.), length of the hospitalization, admission status (voluntary/involuntary), the main diagnosis (organic (F0-G1), substance use (F1), psychotic disorders (F2), bipolar disorder (F30-31), depressive disorders (F32-33) anxious and behavioral disorders (F4-F5), personality disorder (F6), other diagnoses (developmental (F7-F8-F9) and others)). The admission total and item 1 score of the Health of the Nation Outcome Scales (HoNOS) were used to reflect the global burden of symptoms and the level of aggressive behaviour, respectively. HoNOS is a common tool used to measure mental health and social/behavioral functioning. It is a 12-item scale used in all Swiss hospitals to assess the burden of symptoms and the repercussions on inpatients’ mental health and functioning. Each item is scored on a five-level intensity scale (no problem, minor problems, mild/moderately severe, severe, very severe). Higher scores indicate a higher burden of symptoms. It’s also useful to monitor and improve the quality and effectiveness of mental health services (18).

#### 2.2.2. Forced medication, according to the Article 434 of the Civil Code

The following variables were also retrieved from the patients’ medical records and analyzed: the reason for this kind of forced medication [aggressiveness toward others, self-harm (suicidal or self-aggressive behavior) or risk of endangerment to self (clinical and psycho-social endangerment not related to suicidal or aggressive behavior)], intramuscular injection (yes/no), appeal to article 434 of the Swiss Civil Code (yes/no), appeal accepted by the Civil Court (yes/no), immediate initiation of treatment (yes/no), other coercive measures, clinical course after this medical decision. We categorized the clinical course into five possible issues based on the patients’ electronic files: positive course with recovery, change of medication because ineffective, redaction of another Article 434 in case of no response and patient’s refusal, complex situations without evolution, and transfer to forensic psychiatry.

### 2.3. Data analysis

Descriptive analyses of the sample were conducted comparing patients treated against their will according to art. 434 and patients who were not subject to this kind of coercion (no forced medication or emergency forced medication). *T*-test and Pearson’s chi-square test were used to compare continuous, respectively, categorical variables.

A logistic regression analysis was performed to analyze the factors influencing the use of involuntary treatment. The experience of involuntary treatment, according to article 434 (yes/no), was used as the dependent variable. Following predictors were included in the regression, based on univariate group comparisons (threshold set at *p* < 0.05) and a previous analysis investigating the factors influencing the use of other coercive measures (19): age, gender (f/m), diagnostic category (categorized as psychotic disorders vs. other diagnoses), admission mode (voluntary/involuntary), HoNOS item 1 score at admission. Predictors were entered in the regression using an enter method.

Significance was set at *p* < 0.05. Analyses were performed using SPSS 25.

## 3. Results

### 3.1. Descriptive analyses

#### 3.1.1. Patient characteristics

Between 2018 and 2021, 71 out of 4,326 patients (1.64%) underwent medical treatment according to article 434 of the Swiss Civil Code during their stay in a psychiatric hospital. As to patients’ characteristics, 25 of them (35.2%) were male [control group: 2115 (49.7%), *p* = 0.015]. Age was not significantly different (mean 43.2; SD ± 11.56 in the forced medication group vs.42.34; SD ± 11.92 in the control group). Sixty-five patients (91.5%) who underwent forced medication were involuntarily admitted vs. 1790 (42.1%) in the control group (*p* < 0.01). Both groups significantly differed regarding the main diagnosis (*p* < 0.001) (Table 1).

**Table 1:** Descriptive analyses.

Patient-related and clinical variables	No Art. 434	Art 434	Test	df	Value of *p*
*N* = 4,326 (%)	4,255 (98.35)	71 (1.64)			
Gender = male (%)	2,115 (49.7)	25 (35.2)	5.871^1^	1	0.015*
Age [mean (SD)]	42.3 (11.92)	43.2 (11.56)	−0.598^2^	4,324	0.55
Involuntary admission [*N* (%)]	1790 (42.1)	65 (91.5)	69.808^1^	1	<0.001*
Main Diagnosis [*N* (%)]			61.314^1^	4	<0.001*
F2 psychotic disorder	1835 (43.1)	63 (88.7)			
F31 bipolar disorder	600 (14.1)	6 (8.5)			
F3 depressive and anxious disorders	914 (21.5)	0 (0)			
F6 personality disorder	527 (12.4)	1 (1.4)			
Others	379 (8.9)	1 (1.4)			
Admission HoNOS [mean (SD)]	24.32 (6.5)	23.39 (7.6)	−1.002^2^	4,072	0.316
Admission HoNOS item 1 [mean (SD)]	2.51 (1.44)	2.07 (1.4)	−2.492^2^	4,242	0.013*

^1^Pearson’s chi-square test. ^2^*T*-test. HoNOS, Health of the Nation Outcome Scales; SD, standard deviation.

#### 3.1.2. HoNOS score

As to the HoNOS scores at admission, there were no significant differences between both groups regarding the total score [mean score 23.39 (± SD 1,44) in the group who received a medication without consent vs. 24.32 (± SD 1,4) in the group without Art.434]. However, patients in the forced treatment group scored significantly lower on item 1 (aggressive behavior) (*p* = 0.013) (Table 1).

#### 3.1.3. Course and outcome of forced medication

As to forced medication itself, the analysis showed that the risk of self-endangerment (mostly caused by disorganization) was the most frequent justification [*N* = 38 (53.5%)]. Forty-two patients (59.2%) received solely an oral medication vs. 29 (40.8%) who received intramuscular injections. Thirty-three (46.4%) patients appealed this medical decision, and only four appeals were accepted (12.1%), while 84.4% were rejected by the Civil court. In 58 (74.3%) clinical situations, the involuntary medication was administered without waiting for the legal deadline to appeal to the court. As to additional coercive measures, seclusion was experimented by 25 (35.2%) patients.

About the clinical course of patients who received involuntary treatment, most of them (70.4%) recovered after the forced medication. In 15.4% of cases, treatment has been changed (with the patient’s agreement) because of a lack of effectiveness. Seven patients were subjected to a second forced medication decision because the first treatment was not successful, and they still refused treatment (Table 2).

**Table 2:** Characteristics and outcome of forced medication under art. 434.

	*N*	%
Reason of medication under Article 434 of the Swiss Civil Code:		
Aggressiveness toward others	32	45.10%
Self-harm	1	1.44%
Risk of endangerment to self	38	53.50%
Intramuscular injection medication		
Yes	42	59.20%
No	29	40.80%
Appeal to article 434		
Yes	33	46.40%
No	38	53.50%
Appeal accepted		
Yes	4	12.10%
No	28	84.80%
Withdrawn	1	3%
Waiting time-limit appeal before medication:		
Yes	20	28.10%
No	51	71.80%
Others coercive measures:		
Seclusion	25	35.20%
Mechanical restraint	1	1.40%
Clinical course:		
Positive	50	70.40%
Change of treatment	11	15.40%
New article 434	7	9.85%
Complex situation without evolution	2	2.80%
Transfer to forensic psychiatry	1	1.40%

### 3.2. Multivariable analyses

The performed logistic regression analysis showed that both gender, admission mode, and main diagnosis are significant predictors of involuntary treatment. Compared to men, women were at higher risk of being treated without consent [Odds Ratio (OR) 2.22; 95% CI 1.31–3.74]. Similarly, patients with a diagnosis of psychotic disorder were about 13 times more at risk of being involuntarily treated (OR 7.84; 95% CI 3.71–16.55) compared to any other main diagnosis. Being involuntarily admitted was also correlated with a higher risk of being subject to forced medication under Article 434 (OR 10.95; 95% CI 4.64–25.82). Neither age nor the level of aggressive behaviour as measured by the HoNOS item 1 score at admission were significant risk factors for this kind of involuntary treatment (Table 3).

**Table 3:** Results of the logistic regression analysis.

	*B*	OR	95% CI	
Age	0.002	1.002	0.980	1.024
Female gender	0.795	2.215*	1.313	3.738
Involuntary admission	2.393	10.946*	4.641	25.816
HoNOS item 1	−0.024	0.976	0.815	1.168
Psychotic disorder	2.059	7.840*	3.713	16.552

OR, odds ratio; 95% CI, 95% confidence interval; HoNOS, Health of the Nations Outcome Scale; *p < 0.005.

## 4. Discussion

The goal of our study was to analyze factors influencing the use of forced medication outside of emergencies, which is regulated by Article 434 of the Swiss Civil Code. Between January 1, 2018, and December 31, 2021, 71 (1.64%) adult patients were subjected to such an involuntary medication during their hospital stay. Concerning demographic risk factors, female gender was associated with a higher risk of this type of coercion, while age did not represent a significant risk. As to clinical risk factors, the incidence of forced treatment was strongly associated with a diagnosis of psychotic disorder, as was involuntary hospital admission. Neither HoNOS global score nor item 1 HoNOS score were significant predictors of this coercive measure (Tables 1, 2).

Many articles report a wide range of coercion use between 0.4 and 66% of hospitalized patients with notable variations between countries and hospitals (3, 20–22). This disparity shows that coercion is a very heterogeneous phenomenon worldwide and should make us reflect deeply on ethical and clinical challenges in mental health. In addition, the use of coercion is not only linked to clinical factors but also to institutional and cultural aspects. This can explain the wide range reported in different research works. A study in 10 European countries revealed that forced medication represented a percentage between 31 and 81% of all coercive measures (23). But, once again, these studies do not distinguish coercion in emergency situations from non-urgent decisions. In our study, we can consider the prevalence of 1.64% among psychiatric inpatients subjected to forced treatment, as analyzed in the present study, as being a subgroup of forced medicated patients. Switzerland is, in fact, one of the few countries to have a specific law for forced medication, allowing to distinguish between both kinds of coerced treatment. Compared to a previous study analyzing the use of coercive measures in the same hospital, 16.4% were subject to any coercive measures, including seclusion, mechanical restraint, or any forced medication. Among these patients, seclusion was the most used measure (90.8%) and was associated with younger age, aggressive behavior, male gender, and a diagnosis of a psychotic disorder (19). Forced medication under Article 434 thus concerns only a small number of patients with a specific clinical profile.

In fact, by contrast with other studies, women in our sample were at higher risk of being subjected to involuntary treatment because of self-endangerment (24–26). Indeed, endangerment to self is not referring to the risk of suicide but to the psycho-social consequences of psychosis. A hypothesis could be that men have more often aggressive behavior, which is frequently managed with other coercive interventions (seclusion, medication, etc.) in an emergency instead of by a longer forced treatment (27). According to a study on staff views and emotions, patients’ endangerment of others considerably raises the likelihood of the application of coercive measures (28).

Like other studies, we did not find a correlation between age and involuntary treatment (21, 26). A larger group of patients would be useful to further analyze this correlation.

The association between involuntary hospital admission and forced medication is not surprising, as involuntary admission indicates a difficult and complex clinical situation as well as a lack of consent to engage in psychiatric care. Previous research consistently showed that involuntary admission is correlated with coercive measures in general (19, 29).

Patients suffering from a psychotic disorder are significantly more at risk of being subject to involuntary treatment. This finding is in line with Luciano et al. critical review of the use of coercion in mental health settings and with other articles (16, 30, 31). Denial is the main characteristic of psychosis. Because of the poor insight, patients suffering from a psychotic disorder do not often recognize to have a mental illness and consequently are not compliant with treatment (32). Psychiatric patients with poor insight who do not consent to treatment often have a bad prognosis, which could explain the correlation to a higher risk of involuntary treatment during hospitalization (33). In addition, positive symptoms cause a different perception of reality, while negative symptoms induce the patient to neglect himself. This could explain the non-compliance, too, with deleterious consequences on health and social inclusion (34, 35). For all these reasons, these kinds of inpatients are more likely to receive prolonged forced medication during their hospital stay. In fact, they risk psychosocial deterioration, even if they do not present imminent aggression or danger.

As shown in the results, patients receiving treatment by Article 434 presented a lower level of aggression at admission compared to other patients. This is particularly interesting as a previous investigation showed that item 1 score at admission significantly correlates with seclusion (19). This strengthens the idea that this kind of coerced treatment concerns a specific patient subgroup and is not related to violent behavior or endangerment of others. Indeed, the main reason (38%) for this type of involuntary treatment is the risk of endangerment to self (Table 2).

When considering coercive measures, inpatient services thus seem to be confronted with two different groups of patients: on one side, a group of agitated male patients with aggressive behavior – usually treated in emergency and subject to seclusion – and on the other side, non-agitated female patients with chronic psychosis, endangering themselves and facing very severe consequences for their health and social situation. This calls for specific interventions aiming to reduce the use of forced medication. Several studies focus on the balance between protection and coercion and try to understand how coercion can be avoided (36, 37). Nonetheless, there are no specific studies investigating prevention methods that could avoid forced medication. A deeper knowledge of the reasons justifying the refusal of treatment would be extremely useful to implement preventive strategies. Studies on the prospects of coercion perception by patients and caregivers are lacking, as well. The use of advanced directives or joint crisis plans could be regarded as promising to help patient state their will and preferences regarding treatment and crisis management. The implementation of assisted decision-making tools could also be a way to promote empowerment and overcome fears and reluctance about psychopharmacological treatments, also to strengthen adhesion to treatment in the outpatient setting. As 70.4% of patients subjected to this coercion measure could recover and leave the hospital, follow-up investigations, both quantitative and qualitative, would be useful to know the long-term benefit of this kind of forced medication.

Finally, results also show that 53.5% of patients who received forced medication did not appeal the decision. This might be explained by a patient’s disorganization during the decompensation, a miscomprehension of the situation, or a lack of communication between patients and staff. Further qualitative investigations are needed to explore our hypothesis. Of 33 appeals, only four (12.1%) were accepted by the civil court, while 84.4% were refused, and the patient received involuntary treatment. This data supports that repeated medication was necessary in most cases. A broader reflection on inpatients’ rights and legal needs should be considered, especially to help patients know about and understand their rights. The inclusion of relatives and legal bystanders should be an integral part of inpatient care in this regard. A broader reflection on the notion of endangerment and risk for oneself should also be conducted with all stakeholders, including representatives of the justice system.

### 4.1. Strengths and limitations

This research builds on previous studies about coercion carried out in the Geneva University Hospitals, here focusing specifically on medication regulated by Article 434 of the Swiss Civil Code (3, 19, 38). This is the first study on this subject, and it could be a foundation for further reflection. As this specific forced medication is a rare event, a larger sample would be needed to include more potential predictors in the analysis. More socio-demographic data could also be useful to better identify the patient profile most at risk and to potentially develop specific prevention strategies. Another important limitation is the lack of information about the reasons for inpatients’ refusal of treatment. This data would be useful to promote compliance and adherence to care. We could also better investigate how clinical situations evolve and follow up patients after discharge to understand if there is a long-term benefit in this measure of coercion.

## 5. Conclusion

Between 2018 and 2021, 71 inpatients were subjected to forced medication under Article 434 of the Swiss Civil Code. Most of them were women and suffered from psychotic disorders. The biggest reason for this measure of coercion was the risk of self-endangerment. Psychotic denial, poor insight, and a different perception of reality may induce patients to neglect themselves. We should focus on this specific subgroup of patients to create a better care network and minimize the deterioration in patients’ psychosocial functioning. We should be more attentive to behavioral disorganization and identify the first signs of decompensation more quickly. Specific prevention strategies should thus be developed, including early crisis interventions in the community, the use of advanced directives or crisis plans, or the implementation of decision-making tools.

As to legal aspects, inpatients should be better informed about their rights and supported during this process by their relatives or appointed legal bystanders. Better cooperation with associations representing patients could be helpful in this matter.

## Data availability statement

The raw data supporting the conclusions of this article will be made available by the authors, without undue reservation.

## Author contributions

GM was involved in data collection, curation of the database, data analysis and drafting of the manuscript. OS and SK contributed to study design and revision of the manuscript. AW was involved in study conception and design, data analysis and manuscript revision. All authors read, and approved the submitted version.

## Funding

Open access funding by University of Geneva.

## Conflict of interest

The authors declare that the research was conducted in the absence of any commercial or financial relationships that could be construed as a potential conflict of interest.

## Publisher’s note

All claims expressed in this article are solely those of the authors and do not necessarily represent those of their affiliated organizations, or those of the publisher, the editors and the reviewers. Any product that may be evaluated in this article, or claim that may be made by its manufacturer, is not guaranteed or endorsed by the publisher.

## References

[1] Pignon B Rolland B Tebeka S Zouitina-Lietaert N Cottencin O Vaiva G. Critères de soins psychiatriques sans consentement. Revue de littérature et synthèse des différentes recommandations. Presse Med. (2014) 43:1195–205. doi: 10.1016/j.lpm.2014.02.032, PMID: 25081393

[2] Kallert TW. Coercion in psychiatry. Curr Opin Psychiatry. (2008) 21:485–9. doi: 10.1097/YCO.0b013e328305e49f 18650692

[3] Chieze M Hurst S Kaiser S Sentissi O. Effects of seclusion and restraint in adult psychiatry: a systematic review. Front Psych. (2019) 10:491. doi: 10.3389/fpsyt.2019.00491, PMID: 31404294 PMC6673758

[4] Sagduyu K Hornstra R Munro S Bruce-Wolfe V. A comparison of the restraint and seclusion experiences of patients with schizophrenia or other psychotic disorders. Mo Med. (1995) 92:303–7. PMID: 7643843

[5] Tooke SK Brown JS. Perceptions of seclusion: comparing patient and staff reactions. SLACK Incorporated: Thorofare, NJ; (1992). 30, 23–26, doi: 10.3928/0279-3695-19920801-09 1512737

[6] Richardson BK. Psychiatric inpatients' perceptions of the seclusion-room experience. Nurs Res. (1987) 36:234–8. doi: 10.1097/00006199-198707000-00012, PMID: 3648697

[7] Meehan T Bergen H Fjeldsoe K. Staff and patient perceptions of seclusion: has anything changed? J Adv Nurs. (2004) 47:33–8. doi: 10.1111/j.1365-2648.2004.03062.x, PMID: 15186465

[8] van der Merwe M Muir-Cochrane E Jones J Tziggili M Bowers L. Improving seclusion practice: implications of a review of staff and patient views. J Psychiatr Ment Health Nurs. (2013) 20:203–15. doi: 10.1111/j.1365-2850.2012.01903.x, PMID: 22805615

[9] Kallert TW Glöckner M Onchev G Raboch J Karastergiou A Solomon Z. The EUNOMIA project on coercion in psychiatry: study design and preliminary data. World Psychiatry. (2005) 4:168–72. PMID: 16633543 PMC1414770

[10] Sheehan KA. Compulsory treatment in psychiatry. Curr Opin Psychiatry. (2009) 22:582–6. doi: 10.1097/YCO.0b013e328330cd15 19654545

[11] Brown R Martin D Hickman N Barber P. Mental health law in England and Wales: A guide for mental health professionals Sage (2023).

[12] Zielasek J Gaebel W. Mental health law in Germany. BJPsych Int. (2015) 12:14–6. doi: 10.1192/S2056474000000088, PMID: 29093837 PMC5619597

[13] Flammer E Steinert T. Involuntary medication, seclusion, and restraint in German psychiatric hospitals after the adoption of legislation in 2013. Front Psych. (2015) 6:153. doi: 10.3389/fpsyt.2015.00153 PMC4623390 26578985

[14] Dupont M Laguerre A Volpe A. Soins sans consentement en psychiatrie. Comprendre pour bien traiter Rennes: Presses de l'EHESP (2015).

[15] Matta D Carta SK Spagnesi A Medda S Graziano M. Aspetti medico-legali e giuridico-deontologici del trattamento sanitario obbligatorio. Pratica Medica Aspetti Legali. (2008) 2:175–9. doi: 10.7175/pmeal.v2i4.403

[16] Luciano M Sampogna G Del Vecchio V Pingani L Palumbo C De Rosa C. Use of coercive measures in mental health practice and its impact on outcome: a critical review. Expert Rev Neurother. (2014) 14:131–41. doi: 10.1586/14737175.2014.874286, PMID: 24382132

[17] Jarrett M Bowers L Simpson A. Coerced medication in psychiatric inpatient care: literature review. J Adv Nurs. (2008) 64:538–48. doi: 10.1111/j.1365-2648.2008.04832.x, PMID: 19120567

[18] Pirkis JE Burgess PM Kirk PK Dodson S Coombs TJ Williamson MK. A review of the psychometric properties of the health of the nation outcome scales (HoNOS) family of measures. Health Qual Life Outcomes. (2005) 3:1–12. doi: 10.1186/1477-7525-3-76, PMID: 16313678 PMC1315350

[19] Chieze M Courvoisier D Kaiser S Wullschleger A Hurst S Bardet-Blochet A. Prevalence and risk factors for seclusion and restraint at Geneva’s adult psychiatric hospital in 2017. Eur. J. Psychiatry. (2021) 35:24–32. doi: 10.1016/j.ejpsy.2020.06.006

[20] Mann K Gröschel S Singer S Breitmaier J Claus S Fani M. Evaluation of coercive measures in different psychiatric hospitals: the impact of institutional characteristics. BMC Psychiatry. (2021) 21:1–11. doi: 10.1186/s12888-021-03410-z 34419009 PMC8380405

[21] Hotzy F Mötteli S Theodoridou A Schneeberger AR Seifritz E Hoff P. Clinical course and prevalence of coercive measures: an observational study among involuntarily hospitalised psychiatric patients. Swiss Med Wkly. (2018) 148:w14616. doi: 10.4414/smw.2018.14616 29698543

[22] Beghi M Peroni F Gabola P Rossetti A Cornaggia CM. Prevalence and risk factors for the use of restraint in psychiatry: a systematic review. Riv Psichiatr. (2013) 48:10–22. doi: 10.1708/1228.13611, PMID: 23438697

[23] Raboch J Kališová L Nawka A Kitzlerová E Onchev G Karastergiou A. Use of coercive measures during involuntary hospitalization: findings from ten European countries. Psychiatr Serv. (2010) 61:1012–7. doi: 10.1176/ps.2010.61.10.1012, PMID: 20889640

[24] Pawlowski T Baranowski P. How patients’ characteristics influence the use of coercive measures. Indian J Psychiatry. (2017) 59:429–34. doi: 10.4103/psychiatry.IndianJPsychiatry_100_17, PMID: 29497184 PMC5806321

[25] Bakola M Peritogiannis V Stuckler D Kitsou KS Gourzis P Hyphantis T. Who is coercively admitted to psychiatric wards? Epidemiological analysis of inpatient records of involuntary psychiatric admissions to a university general Hospital in Greece for the years 2008–2017. Int J Soc Psychiatry. (2023) 69:267–76. doi: 10.1177/00207640221081793, PMID: 35232289

[26] Flammer E Steinert T Eisele F Bergk J Uhlmann C. Who is subjected to coercive measures as a psychiatric inpatient? A multi-level analysis. Clin Pract Epidemiol Mental Health. (2013) 9:110–9. doi: 10.2174/1745017901309010110, PMID: 23986786 PMC3744855

[27] Schuwey-Hayoz A Needham I. Caractéristiques de l’agressivité des patients dans un hôpital psychiatrique en Suisse. Rech Soins Infirm. (2006) 86:108–15. doi: 10.3917/rsi.086.0108 17020242

[28] Krieger E Moritz S Lincoln TM Fischer R Nagel M. Coercion in psychiatry: a cross-sectional study on staff views and emotions. J Psychiatr Ment Health Nurs. (2021) 28:149–62. doi: 10.1111/jpm.12643 32348607

[29] Müller M Brackmann N Jäger M Theodoridou A Vetter S Seifritz E. Predicting coercion during the course of psychiatric hospitalizations. Eur Psychiatry. (2023) 66:e22. doi: 10.1192/j.eurpsy.2023.3, PMID: 36700423 PMC9981454

[30] Janssen W van de Sande R Noorthoorn E Nijman H Bowers L Mulder C. Methodological issues in monitoring the use of coercive measures. Int J Law Psychiatry. (2011) 34:429–38. doi: 10.1016/j.ijlp.2011.10.008 22079087

[31] Hirsch S Thilo N Steinert T Flammer E. Patients’ perception of coercion with respect to antipsychotic treatment of psychotic disorders and its predictors. Soc Psychiatry Psychiatr Epidemiol. (2021) 56:1381–8. doi: 10.1007/s00127-021-02083-z, PMID: 33904940 PMC8316198

[32] Cooke M Peters E Kuipers E Kumari V. Disease, deficit or denial? Models of poor insight in psychosis. Acta Psychiatr Scand. (2005) 112:4–17. doi: 10.1111/j.1600-0447.2005.00537.x 15952940

[33] Ruissen A Widdershoven G Meynen G Abma T Van Balkom A. A systematic review of the literature about competence and poor insight. Acta Psychiatr Scand. (2012) 125:103–13. doi: 10.1111/j.1600-0447.2011.01760.x 21902676

[34] Misdrahi D Llorca P Lancon C Bayle F. Compliance in schizophrenia: predictive factors, therapeutical considerations and research implications. L'Encephale. (2002) 28:266–72. 12091789

[35] Iyer SN Malla AK. Intervention précoce pour la psychose: concepts, connaissances actuelles et orientations futures. Sante Ment Que. (2014) 39:201–29. doi: 10.7202/1027840ar, PMID: 25590552

[36] Zaami S Rinaldi R Bersani G Marinelli E. Restraints and seclusion in psychiatry: striking a balance between protection and coercion. Critical overview of international regulations and rulings. Riv Psichiatr. (2020) 55:16–23. doi: 10.1708/3301.32714, PMID: 32051621

[37] Hirsch S Steinert T. Measures to avoid coercion in psychiatry and their efficacy. Dtsch Arztebl Int. (2019) 116:336–43. doi: 10.3238/arztebl.2019.0336, PMID: 31288909 PMC6630163

[38] Chieze M Hurst S Sentissi O. État des lieux des preuves d'efficacité. Swiss Archives Neurol Psychiatry Psychother. (2018) 169:104–13. doi: 10.4414/sanp.2018.00573

